# The assessment of marrow adiposity in type 1 diabetic rabbits through magnetic resonance spectroscopy is linked to bone resorption

**DOI:** 10.3389/fendo.2024.1518656

**Published:** 2025-01-24

**Authors:** Wei Li, Wei Wang, Minlan Zhang, Qi Chen, Fengyi Li, Shaojun Li

**Affiliations:** ^1^ Department of Radiology, Shanghai University of Medicine & Health Sciences Affiliated Zhoupu Hospital, Shanghai, China; ^2^ Department of Laboratory Medicine, Shanghai University of Medicine & Health Sciences Affiliated Zhoupu Hospital, Shanghai, China

**Keywords:** type 1 diabetes mellitus, marrow adiposity, magnetic resonance spectroscopy, trabecular microarchitecture, bone resorption

## Abstract

**Background:**

Enhanced marrow adiposity is frequently linked with a decline in bone density. The underlying mechanisms responsible for bone loss in diabetes are not well understood. In this investigation, we employed an alloxan-induced diabetes rabbit model to unravel the association between marrow fat content and bone resorption, utilizing magnetic resonance spectroscopy.

**Methods:**

Forty 4-month-old male New Zealand rabbits were randomly allocated into two groups: a control group and an alloxan-induced diabetic group, each consisting of 20 rabbits. Biochemical analyses covered plasma glucose, enzyme levels, lipid profiles, blood urea nitrogen, creatinine levels, and markers of bone turnover. Quantification of bone marrow adipose tissue utilized both MR spectroscopy and histological examinations. Dual-energy X-ray absorptiometry and microcomputed tomography were employed to determine bone density and trabecular bone microarchitectures. The expression levels of marrow adipocyte markers (peroxisome proliferator-activated receptor-gamma2, CCAAT/enhancer-binding protein-α, and fatty acid binding protein 4) and markers of bone resorption [tartrate-resistant acid phosphatase (TRACP) and cathepsin K] were assessed using RT-PCR.

**Results:**

Diabetic rabbits exhibited significant increases in marrow fat fraction (MFF) over time (MFF increased by 13.2% at 1.5 months and 24.9% at 3 months relative to baseline conditions, respectively). These changes were accompanied by the deterioration of trabecular microarchitectures. Marrow adipogenesis was evident through a 31.0% increase in adipocyte size, a 60.0% rise in adipocyte number, a 103.3% increase in the percentage of adipocyte area, and elevated mRNA expressions of marrow adipocyte markers. Osteoclast markers (TRACP and cathepsin K RNA and serum TRACP5b levels) were elevated in diabetic rabbits. MFF exhibited a robust correlation with trabecular bone microarchitectures. A significant positive correlation was identified between ΔMFF and serum ΔTRACP5b levels. Moreover, MFF at 3 months showed a strong positive correlation with serum TRACP5b levels (*r* = 0.763), as well as with the mRNA expression of osteoclast markers, including TRACP (*r* = 0.784) and cathepsin K (*r* = 0.659), all with *p <*0.001.

**Conclusions:**

Rabbits with type 1 diabetes experience an expansion of marrow adiposity, and this enhanced marrow adiposity is associated with increased osteoclast activity.

## 1 Introduction

The recognition of diabetic bone fragility is increasingly gaining prominence as a complication in both type 1 and type 2 diabetes (T1D, T2D). Key features of diabetic bone fragility include diminished bone mass in T1D, unbalanced bone turnover, and alterations in microstructure and bone material properties (1). There is a burgeoning interest in exploring the connection between bone marrow fat and overall skeletal wellbeing. The quantity of marrow fat has been found to correlate with bone mineral density (BMD) (2, 3). Metabolic conditions linked to bone deterioration and fractures, such as diabetes mellitus, obesity, and anorexia nervosa, may potentially impact the accumulation of marrow fat (4). The mechanisms contributing to diabetic bone disease are intricate, with a growing focus on unraveling the role of bone marrow adipose tissue (BMAT) as a pathogenic fat depot. Notably, changes in bone marrow adipose tissue are more consistently observed in T2D compared to T1D (1, 5). In T1D, the levels of bone marrow adipose tissue closely resemble those in healthy controls (3, 6–9), albeit with relatively limited research in this area.

In the context of diabetes mellitus, acquiring a more comprehensive understanding of fat storage within bone marrow cells and elucidating the intricate relationship between osteoblasts and adipocytes within the bone marrow niche are imperative. This knowledge is pivotal for unraveling the mechanisms underpinning diabetes- and age-related marrow fat storage, potentially opening avenues for identifying novel therapeutic targets for conditions such as “fatty bone” and osteoporosis (10).

Longitudinal data play a crucial role in establishing the correlation between marrow fat content and skeletal integrity, as well as in disentangling the assessment of effectiveness in cross-sectional studies (11). Given the inconsistent observations of changes in bone marrow adipose tissue in T1D, our study aims to address this issue by dynamically examining the consistent effects of diabetes conditions on marrow fat content. Conducted over a 3-month period, our longitudinal study employs magnetic resonance spectroscopy in an alloxan-induced diabetes rabbit model, aiming to achieve a comprehensive understanding of these alterations.

## 2 Methods

### 2.1 Experimental animals

All animal care and experimental protocols were conducted following the approval of the Animal Care and Use Committee of the Shanghai University of Medicine & Health Sciences Affiliated Zhoupu Hospital. All applicable international, national, and/or institutional guidelines for the care and use of animals were followed (2023-107). All methods are reported in accordance with the ARRIVE guidelines.

The study utilized a cohort of 40 four-month-old male New Zealand white rabbits with weights ranging between 2.5 and 3.0 kg, sourced from Silaike Experimental Animals Co., Ltd. (Shanghai, China), and the animals were individually housed in stainless steel rabbit racks. The room conditions were controlled to maintain a temperature range of 20°C to 22°C, humidity between 40% and 60%, and a 12-h light–dark cycle. The rabbits had unrestricted access to both water and food. Following a 2-week adaptation phase, the animals were randomly allocated into two sets (*n* = 20 per set). The initial set comprised 20 animals with chemically induced type 1 diabetes mellitus, while the second set consisted of 20 control animals matched for age (orthoglycemic).

### 2.2 Induction of experimental diabetes protocol for alloxan monohydrate injection

Weekly weight assessments were conducted on rabbits throughout the study, with recorded measurements. To administer the alloxan injection, rabbits underwent light anesthesia using ketamine hydrochloride at a dosage of 30 mg/kg and xylazine at 3 mg/kg (intramuscularly). Alloxan monohydrate (Sigma Aldrich Chemical, Saint Louis, MO, USA) was dissolved in sterile normal saline to attain a 5% (W/V) concentration. Subsequently, 100 mg/kg of the solution was promptly administered intravenously through the marginal ear vein over a 2-min duration using a 25-gauge butterfly catheter. At 4, 8, and 12 h post-alloxan injection, a subcutaneous administration of 10 mL of 5% (W/V) glucose was carried out. Additionally, an oral solution containing 20% glucose in tap water was made available through a water bottle *ad libitum* for 1–2 days after confirming hypoglycemia (less than 70 mg/dL). This measure was taken to prevent the occurrence of hypoglycemic shock. Rabbits with blood glucose levels consistently below 300 mg/dL for over 1 week following the initial alloxan injection were subjected to a subsequent dose of alloxan (100 mg/kg IV) as described previously (12, 13). This additional administration aimed to sustain a blood glucose level exceeding 300 mg/dL throughout the study period. In cases where morning blood glucose levels exceeded 350 mg/dL, a daily subcutaneous administration of regular insulin (Novolin-R, Novo Nordisk [China] Pharmaceuticals Co. Ltd. Production Plant, Tianjin, China) was implemented using a 30-gauge needle. Blood samples were obtained from the ear marginal vein using a 25-gauge needle for biochemical analysis weekly for 1 month then biweekly until the end of the experiment.

Upon completion of the experiment, bone specimens were promptly collected following the euthanasia of the animals via intravenous administration of an overdose of ketamine hydrochloride. Soft tissue was carefully removed from the bones, after which dual-energy X-ray absorptiometry was utilized to measure *ex-vivo* BMD at the isolated left whole femur and L5 vertebrae. Following BMD assessment, the left femora underwent decalcification for histopathological examination. Meanwhile, the right tibias were harvested for micro-computed tomography (micro-CT) analysis, and the left tibias were allocated for quantitative PCR analysis.

### 2.3 MR imaging and spectroscopic imaging protocol

The rabbits were positioned ventrally, with hind limbs separated from the trunk, for the purpose of scanning the left femur under baseline conditions, as well as at 1.5 and 3 months, all conducted under general anesthesia as previously mentioned (11). *In-vivo* MR spectroscopy data were obtained using a 3-T instrument (MAGNETOM Skyra; Siemens Medical Systems, Erlangen, Germany) equipped with an integrated body coil for signal transmission and a quadrate knee array for signal reception. Prior to acquiring MR spectroscopy data, MR imaging was carried out with a sagittal, coronal, and axial scout T2-weighted fast spin echo sequence specifically targeting the left femur. This step was crucial for accurately defining the spectral acquisition box.

After the completion of the imaging procedure, we obtained single-voxel MR spectroscopy data at the distal femur using a pulse sequence specifically designed for this purpose—single-voxel point-resolved spectroscopy. The parameters for this sequence were meticulously configured: repetition time = 5,000 ms, echo time = 30 ms, 64 averages without water suppression, number of data points = 1,024, voxel size = 6 × 6 × 14 mm³, and receiver bandwidth = 2,000 Hz. For each voxel placement, automated optimization of gradient shimming was systematically conducted. The total duration of the entire acquisition, encompassing both the localizer sequence and bone marrow MR spectroscopy, including the shimming process, averaged approximately 5 min.

The spectral data underwent time-domain processing using the jMRUI software, incorporating the Accurate, Robust, and Efficient Spectral fitting (AMARES) algorithm. During preprocessing, calibration was executed based on the principal –(CH_2_)n– peak at 1.30 ppm, and zero-order automatic phasing was applied. Line widths were unconstrained, and peak frequencies were fine-tuned to be within 0.1 ppm of their theoretical chemical shifts. Quantification involved identifying five peaks, including the water peak at 4.7 ppm and four lipid peaks at 0.9, 1.3, 2.3, and 5.2 ppm, with their assignments referenced from prior studies (14). Post-processing, the amplitudes of the lipid and water peaks were determined. Marrow fat content was computed as the marrow fat fraction (MFF) = [*I*_lipids_/(*I*_lipids_ + *I*_water_)] × 100%, where I_lipids_ represents the cumulative area amplitudes of the resonances at locations 0.9, 1.3, 2.3, and 5.2 ppm, and *I*_water_ denotes the area amplitude of the H_2_O resonance (15).

### 2.4 Biochemical analysis

Plasma was acquired through the centrifugation of blood samples at 3,000 rpm for 20 min at 4°C and subsequently stored at −20°C until analysis. The quantification of glucose, enzymes, lipid profile, blood urea nitrogen, and creatinine levels was measured using automated techniques. Fasting serum insulin levels were determined using a rat insulin enzyme-linked immune absorbent assay kit, adhering strictly to the manufacturer’s instructions. The quantification of HbA1c was carried out utilizing ELISA kits (Nanjing Jianchen Bioengineering Institute, Jiangsu Province, China) in accordance with the protocols provided by the respective manufacturers. The tartrate-resistant acid phosphatase type 5b (TRACP5b) and osteocalcin concentrations were assessed using serum ELISA in accordance with the guidelines provided by the manufacturer (Immunodiagnostic Systems, Inc., East Bolden, UK).

### 2.5 Dual-energy X-ray absorptiometry

We measured the area BMD in the L5 vertebrae and left femur region using the Hologic Discovery Wi dual-energy X-ray absorptiometer Scanner (Hologic Inc., Bedford, MA, USA). To facilitate small animal scanning, we utilized dedicated software, as outlined in prior descriptions (16). The BMD value was automatically calculated, relying on the bone mineral content within the specified measurement region.

### 2.6 Micro-CT

The CT imaging of the right tibiae before they underwent decalcification for sectioning was conducted using a cone-beam X-ray micro-CT system (Healthcare Explore Locus, GE Medical Systems, Milwaukee, USA) with the following settings: X-ray tube voltage = 80 kV, anode current = 80 mA, shutter speed = 3,000 ms, binning factor = 2, angle of increment = 0.5°, and spatial resolution = 19 µm/voxel. Three-dimensional images were reconstructed in 1,024 × 1,024-pixel matrices and analyzed using the MicroView v2.1.2 software program. The specific region of interest analyzed ranged from a slice located 1.8 mm below the growth plate to the 100th distal slice (17). Following thresholding, measurements were taken for trabecular thickness (Tb.Th), trabecular number (Tb.N), trabecular separation (Tb.Sp), bone volume to total volume ratio (BV/TV), structure model index (SMI), and connectivity density (Conn.D). All assessments were conducted in a blinded manner, ensuring no prior knowledge of the sample’s experimental identity during the evaluations.

### 2.7 Hematoxylin and eosin staining

In summary, the left femurs underwent an initial fixation in 10% buffered formalin for 2 days, followed by a 6-week decalcification period with 10% ethylene diamine tetraacetic acid. Subsequently, the specimens were dehydrated using concentrated ethanol, subjected to xylene washing, and ultimately embedded in paraffin wax. The processed femurs were then meticulously sectioned into 5-μm slices along the coronal plane of the distal femora. For the quantification of marrow adipocytes, the sections underwent standard hematoxylin and eosin (H&E) staining. A total of five fields from a single section, each magnified at ×400 per sample, were randomly selected for assessment using a light microscope (Nikon AZ100; Nikon, Tokyo, Japan) and ImageJ software (NIH). The evaluation process encompassed the calculation of adipocyte mean diameter, percentage of adipocyte area, and adipocyte density (adipocyte number per unit bone marrow area, excluding the bone trabeculae) in the analyzed fields, following established protocols (18). Statistical analysis involved determining the mean value of quantitative parameters for marrow adipocytes from all measured fields for each rabbit. Samples were evaluated in a blinded fashion to avoid investigator bias. Given the variability in adipocyte distribution and size within the femur, two researchers independently performed pathological histological assessments to mitigate selection bias. For the final statistical analysis, the mean of their measurements was used to ensure accuracy and reliability.

### 2.8 RNA extraction and quantitative PCR

Total RNA was meticulously extracted from the entire left tibia using TRIzol reagent (Invitrogen, Carlsbad, CA, USA), adhering closely to the manufacturer’s stipulated procedures. The ensuing 20-µL reaction system, composed of 10 µL of FastStart Universal SYBR Green Master (ROX; Roche, Mannheim, Germany), 0.5 µL of forward primers (10 mmol/L), 0.5 µL of reverse primers (10 mmol/L), and 2 µL of complementary DNA, was applied on the ABI 7700 Real-Time PCR System (Applied Biosystems, Mannheim, Germany). Gene-specific primers, meticulously crafted through DNAMAN 9.0, were enlisted for the nuclear receptor peroxisome proliferator-activated receptor-gamma2 (PPARγ2) (forward 5′-GAAAGACAACGGACAAATCACC-3′, reverse 5′-GGGGGTGATATGTTTGAACTTG-3′), CCAAT/enhancer-binding protein-α (C/EBPα) (forward 5′-TGGACAAGAACAGCAACGAG-3′, reverse 5′-TCACTGGTCAACTCCAGCAC-3′), fatty acid binding protein 4 (FABP4) (forward 5′-AAGGTGAAGAGCATCATAACCCT-3′, reverse 5′-TCACGCCTTTCATAACACATTCC-3′), tartrate-resistant acid phosphatase (TRACP) (forward 5′-AAT GCC TCG ACC TGG GA-3′, reverse 5′-CGT AGT CCT TGG CTG CT-3′), cathepsin K (forward 5′-GCA GAG GTGTGT ACT ATG-3′, reverse 5′-GCA GGC GTT GTT CTT ATT-3′), and GAPDH (forward 5′-AGCTTGTCATCAACGGGAAG-3′, reverse 5′-TTTGATGTTAGTGGGGTCTCG-3′). Both standards and samples underwent triplicate runs for robust analysis. Relative quantification, computed as 2^−ΔΔCT^, was determined with GAPDH serving as the steadfast internal control.

### 2.9 Statistical analysis

The data are presented as mean ± standard deviation. Statistical analyses were conducted using SPSS version 25.0 (IBM, Armonk, New York), and significance was determined at a *p*-value less than 0.05. Normality of the data was assessed through the Shapiro–Wilks test. Pearson’s and Spearman’s correlation analyses were employed to explore bivariate associations between variables for normally and non-normally distributed data, respectively. For multiple-time point measurements over time in MFF and serum biomarkers, repeated-measures analysis of variance was employed. Subsequently, the Bonferroni *post-hoc* test was applied to identify differences between groups at each time point. Differences in other studied parameters were assessed using either the Student’s *t*-test or the Mann–Whitney *U* test, depending on the data distribution.

## 3 Results

### 3.1 Changes in body weight and serum biochemical markers

**Figure 1:**
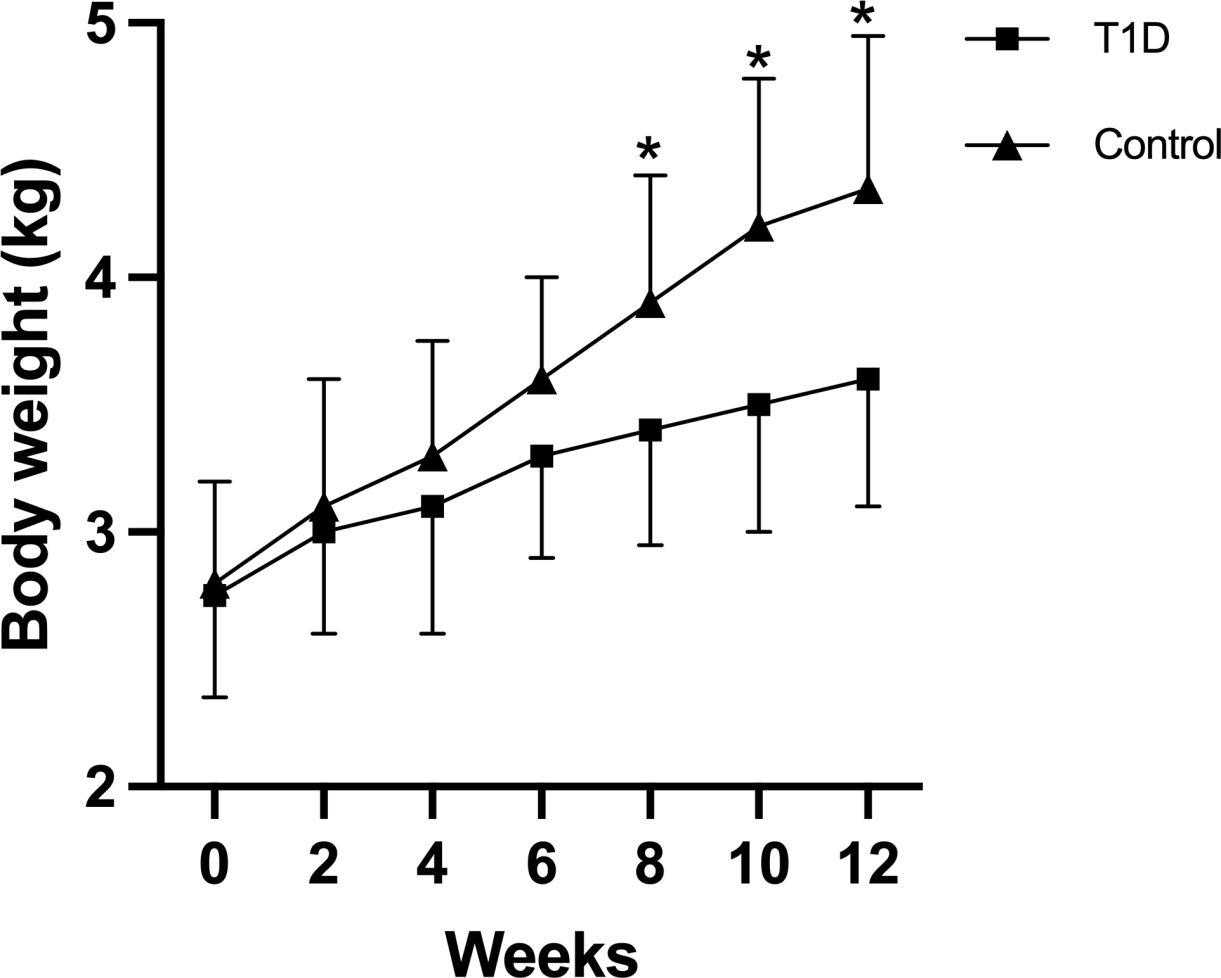
Body weights of rabbits in the two experimental groups were monitored throughout the study period. Data are expressed as mean ± SD [type 1 diabetes (T1D) group, *n* = 19; control, *n* = 19]. *p <0.05 indicates a statistically significant difference between the T1D and control groups. Notably, the increase in body weight was significantly lower in diabetic rabbits compared to the non-diabetic group (*p* < 0.05).

Throughout the duration of this study, 38 out of 40 rabbits (38/40, 95%) successfully completed the entire investigation, while two rabbits unfortunately succumbed to adverse reactions to anesthetics. Despite the significantly elevated blood glucose levels, all diabetic rabbits exhibited a trend toward weight gain. After 5 months, the average final body weight of the diabetic rabbits was 3.60 ± 0.50 kg, compared to 4.35 ± 0.60 kg in the age-matched non-diabetic rabbits. However, the increase in body weight was significantly lower in the diabetic rabbits than in the non-diabetic group (*p* < 0.05) (**Figure 1**). Blood glucose, HbA1C, total cholesterol, triglycerides, and TRACP5b levels showed a time-dependent increase in the T1D group, while insulin and osteocalcin exhibited a time-dependent decrease (**Table 1**). At 3 months, plasma levels of blood urea nitrogen and creatinine were elevated following diabetes induction, although these changes did not reach statistical significance. Notably, diabetic rabbits displayed significantly higher levels of blood glucose, HbA1C, total cholesterol, triglycerides, and TRACP5b compared to normal rabbits at both 1.5 and 3 months. Additionally, a significant reduction in serum insulin and osteocalcin levels was observed in diabetic rabbits compared to their normal counterparts at 3 months.

**Table 1:** Descriptive characteristics of T1D and control rabbits included in the study.

	T1D (*n* = 19)			Controls (*n* = 19)		
	M_0_	M_1.5_	M_3_	M_0_	M_1.5_	M_3_
Blood glucose (mg/dL)	123 ± 17	351 ± 39^a^	370 ± 52^ab^	125 ± 16	123 ± 17*	125 ± 16^#^
Insulin (μIU/mL)	15.8 ± 2.7	10.2 ± 1.9^a^	5.7 ± 1.4^ab^	16.0 ± 2.8	16.3 ± 3.0*	16.1 ± 2.9^#^
HbA1C (%)	5.5 ± 0.6	7.0 ± 0.8^a^	8.5 ± 1.6^ab^	5.4 ± 0.6	5.6 ± 0.7*	5.4 ± 0.5^#^
Total cholesterol (mg/dL)	61.2 ± 6.2	177.5 ± 14.3^a^	211.5 ± 20.9^ab^	62.5 ± 5.9	63.5 ± 5.9*	61.7 ± 6.5^#^
Triglycerides (mg/dL)	127.7 ± 18.0	185.1 ± 27.5^a^	223.8 ± 23.0^ab^	125.3 ± 18.4	129.4 ± 20.7*	127.3 ± 19.8^#^
Blood urea nitrogen (mg/dL)	13.0 ± 1.8	13.5 ± 2.0	14.4 ± 2.1	12.9 ± 1.9	13.2 ± 2.2	12.8 ± 1.7
Serum creatinine (mg/dL)	0.93 ± 0.17	0.95 ± 0.18	1.01 ± 0.20	0.93 ± 0.18	0.91 ± 0.19	0.92 ± 0.18
Serum TRACP5b (U/L)	9.6 ± 1.5	14.3 ± 2.0^a^	16.2 ± 2.8^ab^	9.3 ± 1.3	9.8 ± 1.4*	10.3 ± 1.9^#^
Serum osteocalcin (U/L)	36.1 ± 3.9	35.0 ± 4.3	32.4 ± 5.3^a^	36.8 ± 3.7	38.9 ± 4.5	40.2 ± 6.0^#^

Data are presented as mean ± standard deviation.

HbA1c, hemoglobin A1c; M, month; T1D, type 1 diabetes; TRACP5b, tartrate-resistant acid phosphatase 5b.

a *p* < 0.05 compared to the baseline condition (0 months) and ^b^ *p* < 0.05 compared to 1.5 months in the T1D group.

*p < 0.05 compared to the T1D group at 1.5 months and ^#^ *p* < 0.05 compared to the T1D group at 3 months.

### 3.2 BMD and trabecular bone microarchitecture

In a 3-month study, rabbits in the experimental group exhibited trabecular bone loss compared to the control groups, as depicted in micro-CT images and relevant parameters, exhibiting significant decreases in BV/TV, Tb.Th, and Tb.N but significant increases in Tb.Sp and SMI (**Table 2**). Reductions were also seen in bone mass of the lumbar spine and femur, but did not reach statistical significance. MFF correlated with BV/TV (*r* = –0.802), Tb.Th (*r* = –0.659), Tb.N (*r* = –0.637), Tb.Sp (*r* = 0.834), and SMI (*r* = 0.721) (all *p* < 0.001).

**Table 2:** Bone mass and trabecular microstructure in the two groups of rabbits at 3 months.

Variables	T1D (*n* = 19)	Controls (*n* = 19)	*p*-values
L5 vertebrae BMD (g/cm^2^)	269 ± 40	289 ± 42	0.211
Femur BMD (g/cm^2^)	338 ± 43	354 ± 49	0.313
BV/TV (%)	24.8 ± 4.7	33.0 ± 5.5	<0.001
Tb.Th (*μ*m)	109 ± 20	130 ± 28	<0.001
Tb.N (1/mm)	2.00 ± 0.55	3.09 ± 0.70	<0.001
Tb.Sp (*μ*m)	396 ± 85	231 ± 59	<0.001
SMI	1.62 ± 0.37	0.88 ± 0.20	<0.001

Data are presented as mean ± standard deviation. Comparisons were performed with *t*-test.

BMD, bone mineral density; BV/TV, bone volume/total volume; SMI, structure model index; Tb.N, trabecular number; Tb.Sp, trabecular separation; Tb.Th, trabecular thickness; T1D, type 1 diabetes.

### 3.3 The long bones of rabbits with T1D exhibited a substantial increase in bone marrow adiposity

**Figure 2:**
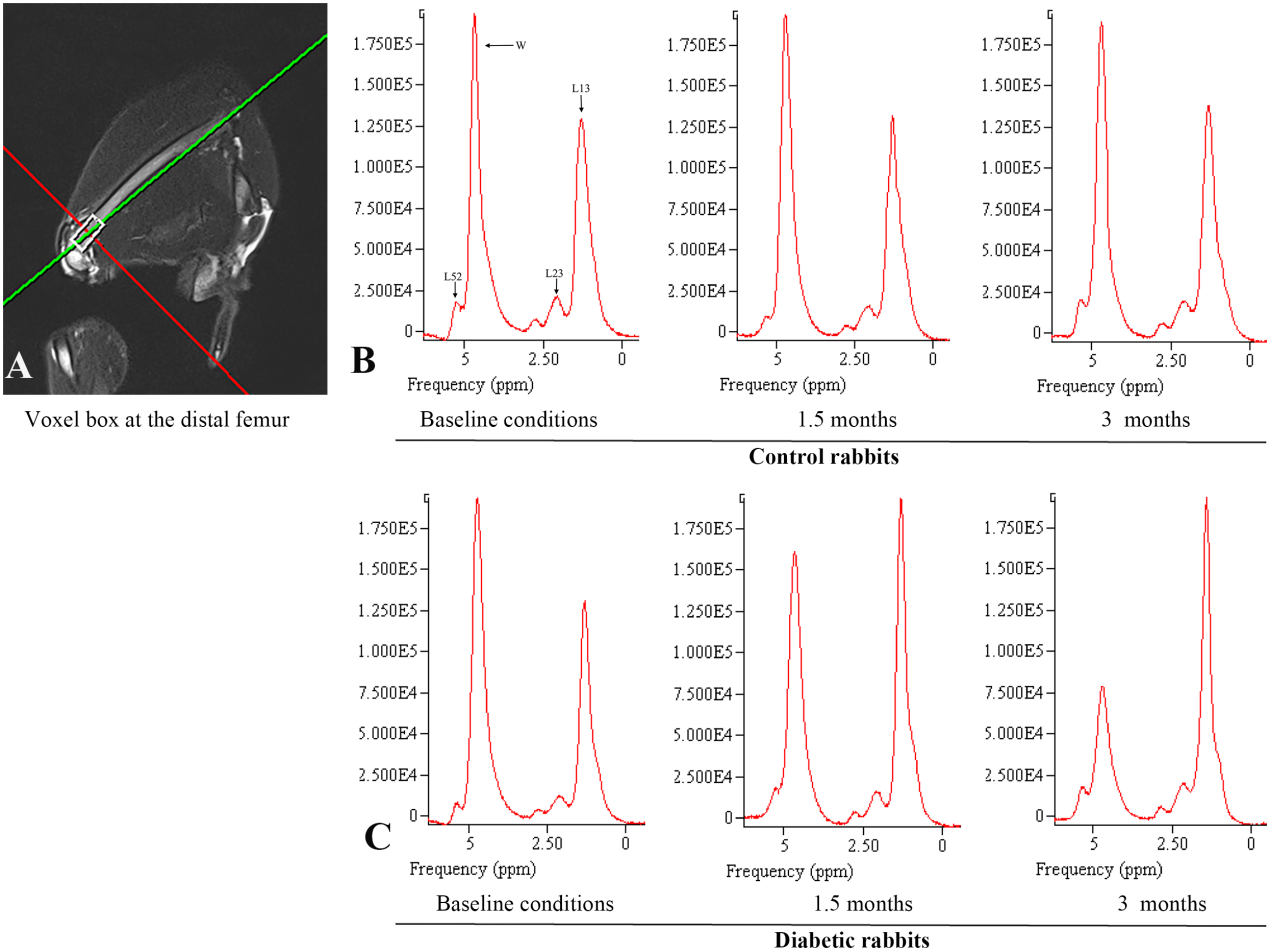
Proton magnetic resonance spectroscopy was employed to analyze the marrow fat fraction of the left femur. **(A)** Single-voxel MR spectroscopy acquisition performed at the distal femur. **(B)** Control rabbits underwent spectroscopic examination at three time points: baseline, 1.5 months, and 3 months. The measured fat fractions were 40.5%, 41.0%, and 41.3%, respectively. **(C)** Diabetic rabbits underwent spectroscopic examination at the same intervals, with fat fractions recorded at 40.2%, 54.9%, and 75.0%, respectively. The identified peaks encompass various proton signals, including bulk CH2 methylene protons (designated as L13 at approximately 1.3 ppm), CH2 methylene protons α- (L23 at approximately 2.3 ppm), water (W at approximately 4.7 ppm), and olefinic protons, which overlap with the glycerol CH methine proton (L52 at approximately 5.2 ppm). Notably, at clinical field strengths (≤3 T MRI), CH3 methyl protons at 0.9 ppm and bulk CH2 methylene protons at 1.3 ppm manifest as a single, consolidated peak.

**Figure 3:**
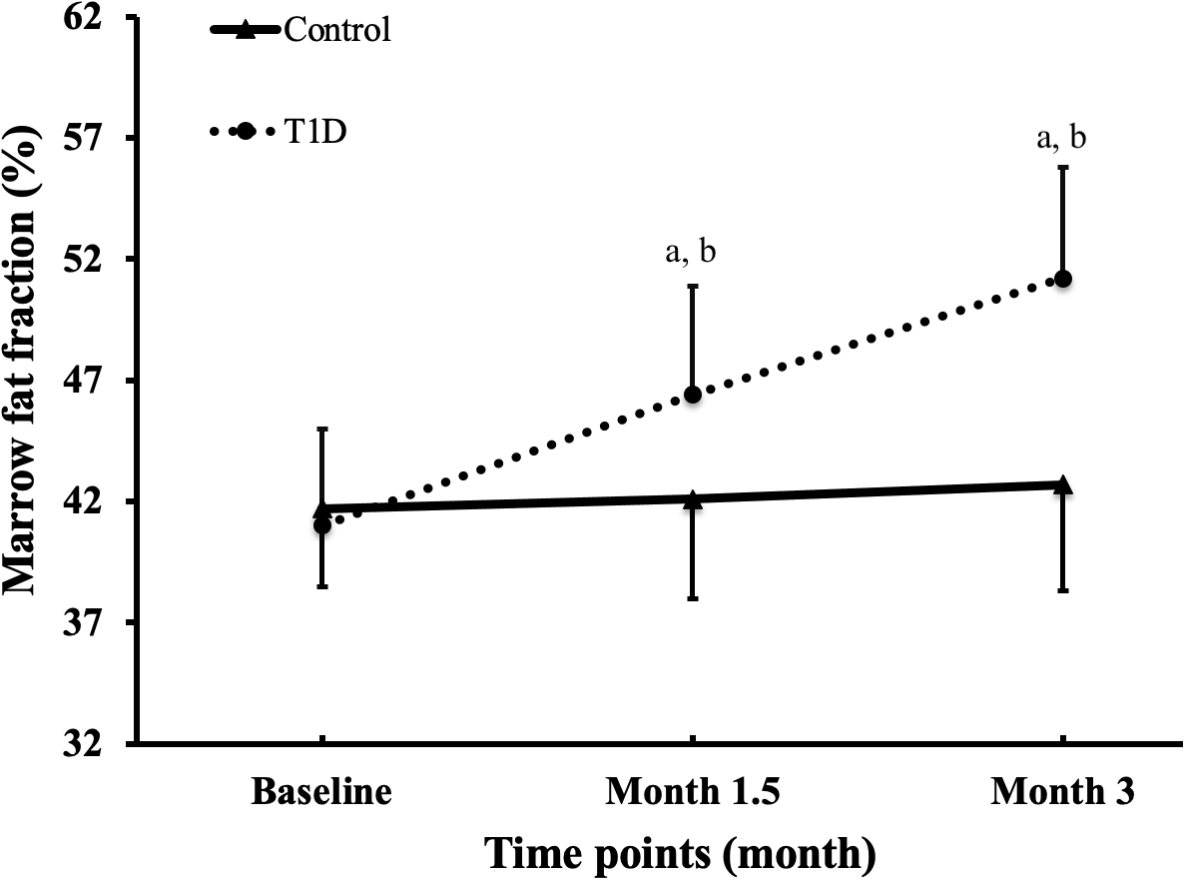
Alterations in marrow fat fraction at the femur were observed across all groups and time points. The data are presented as mean ± SD. ^a^ *p <*0.001 compared to the preceding time point in the type 1 diabetes (T1D) group. ^b^ *p <*0.05 compared to the control group at the corresponding time point.

**Figure 2** is the representative proton MR spectroscopy at the left distal femur monitored at various time points. Changes in MFF of the distal femur are shown in **Figure 3**. Relative to the controls, MFF in the T1D group was increased by 10.2% at 1.5 months and 19.9% at 3 months, respectively. Across various time points, the MFF in diabetic rabbits exhibited a consistent upward trend, rising by 13.2% (*p* < 0.001) at 1.5 months and further increasing by 24.9% at 3 months (*p* < 0.001). No significant temporal changes in MFF were detected in the control group.

**Figure 4:**
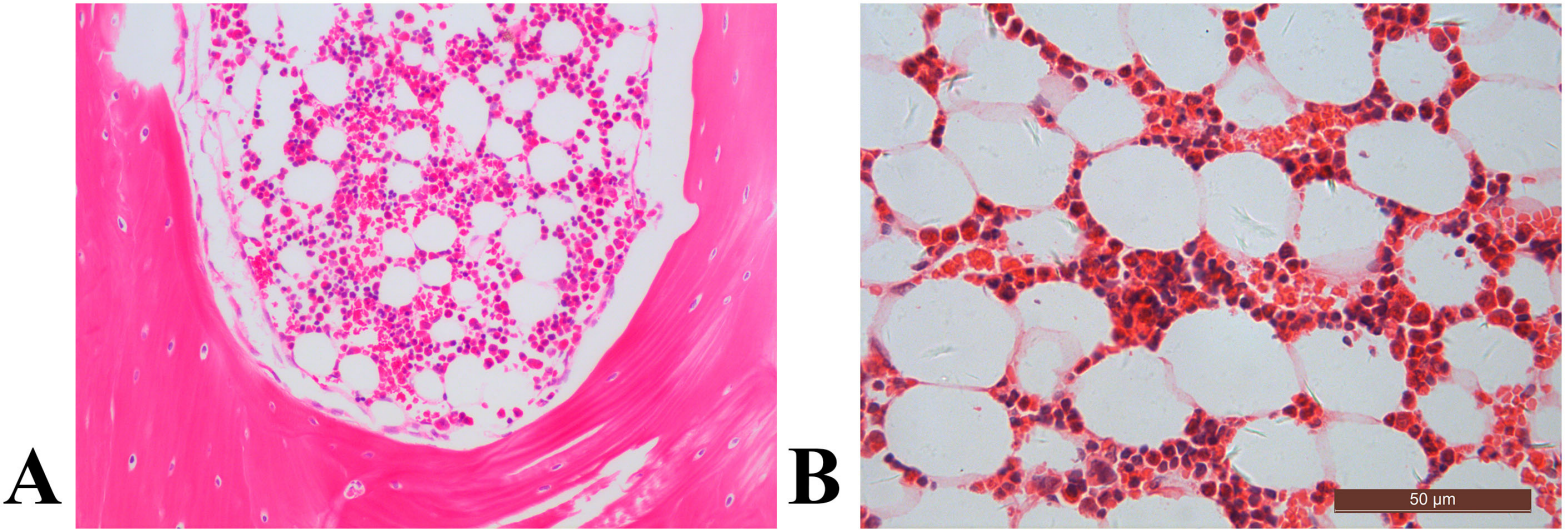
The distal femur, stained with hematoxylin and eosin (original magnification ×400), revealed distinct characteristics. In the control rabbit **(A)**, the bone marrow cavity displayed abundant trabecular bone structures (asterisk), alongside sparse marrow adipocytes (arrow). In contrast, the diabetic rabbit exhibited diminished trabecular bone histopathology, coupled with notable marrow fat accumulation **(B)**.

**Figure 5:**
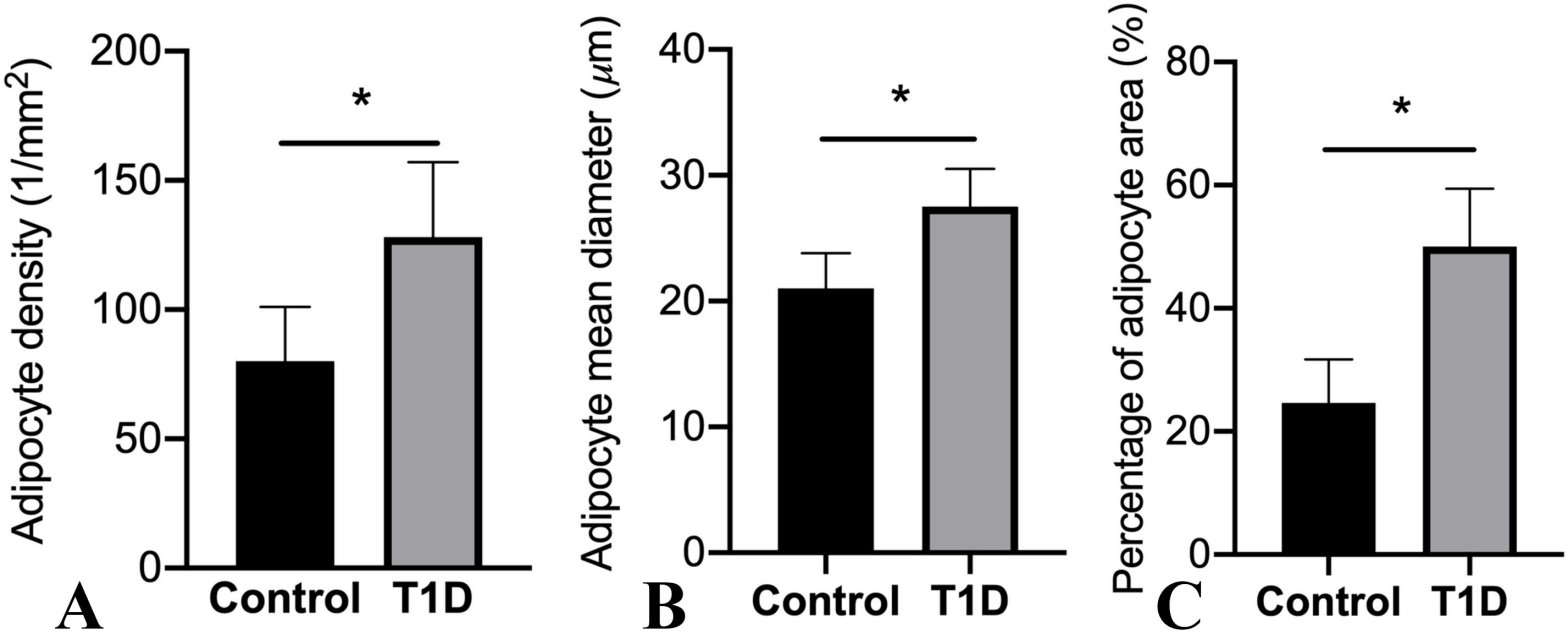
Alterations in marrow adipocyte parameters are depicted. The values in the columns are expressed as mean (SD; *n* = 19 rabbits per group), highlighting significant differences in adipocyte density **(A)**, adipocyte mean diameter **(B)**, and percentage of adipocyte area **(C)** (*p < 0.05) observed among the groups. T1D, type 1 diabetes.

Representative histological images of bone marrow sections revealed a significant augmentation in bone marrow adiposity in T1D rabbits compared to the control group (**Figure 4**). This increase was primarily attributed to increased adipocyte density by 60.0%, elevated adipocyte diameter by 31.0%, and a higher percentage of adipocyte area by 103.3% (**Figure 5**).

**Figure 6:**
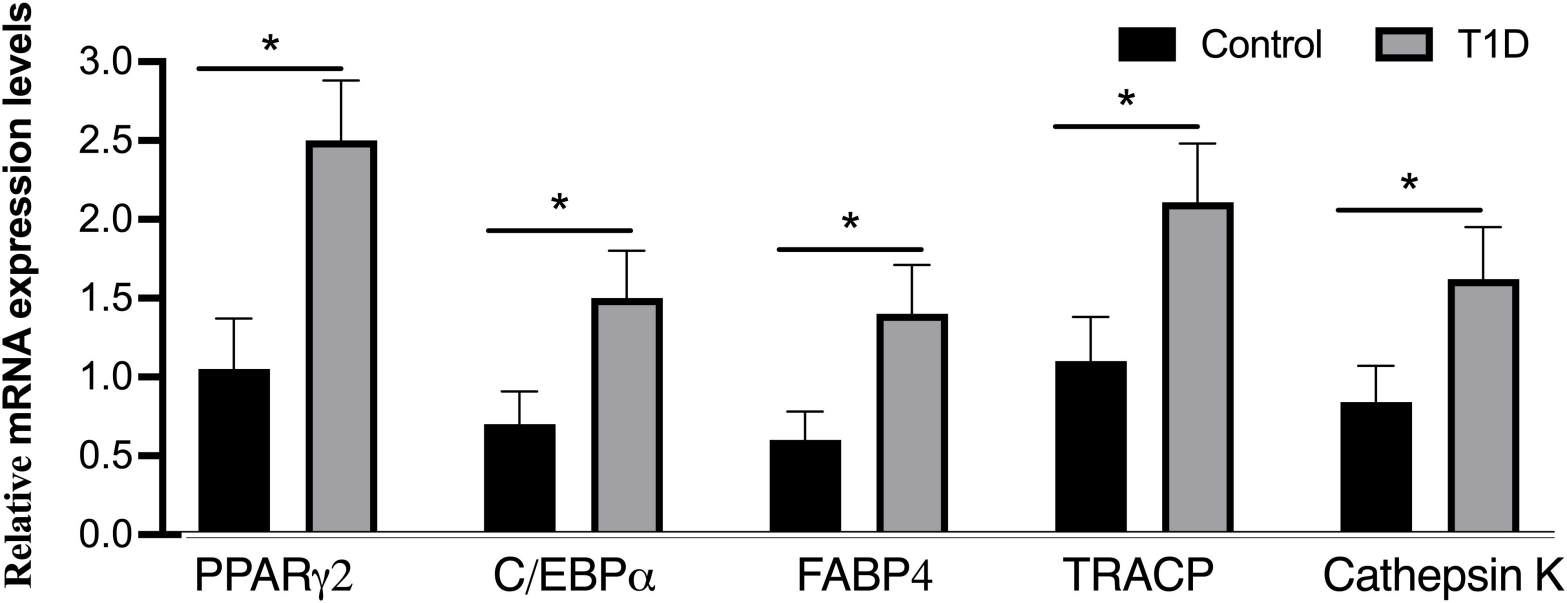
Analysis of mRNA levels for adipogenic markers (PPARγ2, C/EBPα, and FABP4) and osteoclast markers (TRACP and cathepsin K) through real-time PCR. The expression of adipogenic marker genes was evaluated and normalized by GAPDH, with all experiments conducted in triplicate. The presented data represent means ± SD (*n* = 19 per group), revealing significant differences (*p < 0.001) among the groups. C/EBPα, CCAAT/enhancer-binding protein-α; FABP4, fatty acid binding protein 4; PPARγ2, peroxisome proliferator-activated receptor-gamma2; TRACP, tartrate-resistant acid phosphatase.

To delve into the molecular aspects of adipogenic differentiation, we isolated adipocytes from the bone marrow and conducted RT-PCR analyses. Notably, the adipocytic transcription factors, specifically the nuclear receptor PPARγ2 and C/EBPα, along with differentiated adipose cell markers like FABP4, exhibited robust expression levels in the bone marrow of T1D rabbits (**Figure 6**). Importantly, these expression levels were considerably higher than those observed in the control rabbits. Upon closer analysis of gene expression levels in rabbit tibias, it was observed that there is an elevation in the mRNA levels of TRACP and cathepsin K, both recognized as osteoclast markers, in diabetic tibias as opposed to control tibias.

### 3.4 Association of MFF with biochemical markers or osteoclast marker mRNA levels

Using correlation analysis to examine the potential links between marrow adiposity and serum biomarkers or mRNA expression of osteoclast markers, we identified a significant positive correlation between ΔMFF and serum ΔTRACP5b levels, as well as between ΔMFF and serum ΔHbA1C (**Table 3**). Additionally, a significant positive correlation was observed between MFF_M3_ and serum TRACP5b levels (*r* = 0.763, *p* < 0.001), as well as between MFF_M3_ and the mRNA levels of osteoclast markers, including TRACP (*r* = 0.784, *p* < 0.001) and cathepsin K (*r* = 0.659, *p* < 0.001). However, no association was found between ΔMFF and Δosteocalcin, Δblood glucose, or Δinsulin.

**Table 3:** Correlation coefficients between biochemical markers or osteoclast marker mRNA levels in the T1D group.

	ΔTRACP5b		Δosteocalcin		Δblood glucose		Δinsulin		ΔHbA1C		mRNA expression	
	M_1.5–0_	M_3–0_	M_1.5–0_	M_3–0_	M_1.5–0_	M_3–0_	M_1.5–0_	M_3–0_	M_1.5–0_	M_3–0_	TRACP	Cathepsin K
ΔMFF_M1.5–0_	**0.775**	–	0.028	–	0.063	–	0.051	–	**0.384**		–	–
ΔMFF_M3–0_	–	**0.816**	–	0.032	–	0.080	–	0.042	–	**0.411**	–	–
MFF_M3_	–	–	–	–	–	–	–	–	–	–	**0.784**	**0.659**

Significant *p*-values are highlighted in bold. ΔMFF represents the percentage change in MFF between two consecutive time points. The percentage change for the M_1.5–0_ period was calculated using the formula: (MFF_M1.5_ − MFF_M0_)/MFF_M0_ × 100%. This calculation was performed for each time period.

HbA1c, hemoglobin A1c; M, month; MFF, marrow fat fraction; T1D, type 1 diabetes; TRACP, tartrate-resistant acid phosphatase.

## 4 Discussion

Studies conducted on both mice and humans subjected to a high-fat diet have shown an increase in BMAT. This increase was assessed through measurements of BMAT volume, as well as the size and number of bone marrow adipocytes (19–22). Notably, the relationship between BMAT and BMD varied, displaying patterns of increase, stability, or decrease. These variations depended on the type of diabetic model studied, specifically T1D or T2D (5). However, irrespective of the BMD outcomes, the heightened presence of BMAT under high-fat diet conditions was consistently linked to an elevated risk of fractures.

In our study, the research findings highlight a pronounced increase in bone marrow adiposity in T1D rabbits, as evidenced by histological and molecular analyses. The elevated expression of key adipogenic transcription factors and markers underscores the heightened adipogenic differentiation at the molecular level in the bone marrow of T1D rabbits compared to their non-diabetic counterparts. Several studies have highlighted a considerable elevation in BMAT among type 1 diabetic rodents (23, 24), revealing a significant negative correlation between bone formation and the adipocyte proportion within the bone marrow. Furthermore, it has been clarified that diabetic conditions induce a redirection in the differentiation of bone marrow mesenchymal stem cells, favoring the adipogenic lineage over the osteoblastic lineage (24).

Animal studies suggest that individuals with T1D exhibit elevated levels of BMAT (25, 26). However, human studies provide a different perspective, indicating that BMAT levels are comparable between individuals with and without T1D (3, 6–9). While three studies did observe higher BMAT in T1D subjects compared to controls, the differences were not statistically significant. For example, Abdalrahaman et al. (8) reported vertebral BMAT levels of 31.3% in women with T1D and 26.3% in controls (*p* = 0.20), underscoring the lack of statistical significance. A critical limitation of the human studies is their relatively small sample sizes and focus on younger populations, with mean ages ranging from 13 to 40 years (7, 9). Furthermore, some participants with T1D had already received treatment, which may have introduced bias into the findings. Despite these limitations, a recent study reported that individuals newly diagnosed with T1DM exhibit an expansion of marrow fat, and this increased marrow fat content is associated with MRI-based trabecular microstructure changes (27). These results align with our findings, suggesting that newly diagnosed cases may provide clearer insights into marrow fat dynamics. The disparities between animal and human studies may stem from a more pronounced phenotype observed in T1D mouse models, potentially amplifying differences in BMAT levels. Understanding these variations is crucial for reconciling findings across species and advancing the study of bone health in T1D.

Differentiation of bone marrow-derived stromal cells is affected under diabetic conditions (28). At the cellular level, elevated glucose levels can stimulate adipogenic differentiation while suppressing the osteogenic differentiation of these stromal cells. Mechanistically, at the molecular level, these effects are mediated by the regulation of mRNA and protein expression of key factors such as PPARγ, C/EBPβ, and FABP4 (29). The compromised osteogenesis observed in diabetic bone marrow-derived stromal cells is concurrent with a heightened propensity for adipogenic differentiation during culture induction, aligning with existing literature documenting increased bone marrow adiposity in rodents with T1D (25, 26).

The relationship between marrow fat content and insulin resistance has been inconsistently reported across studies. Some studies have demonstrated a positive correlation between marrow fat expansion and insulin resistance (30, 31), while others have found no such association (32, 33). Notably, one study reported a negative relationship between marrow fat content and both fasting insulin levels and insulin resistance in premenopausal women, regardless of their obesity status (32). Conversely, other investigations failed to identify any significant associations. For example, Bani Hassan et al. (34) found no link between marrow fat content and serum glucose, inflammatory markers, or insulin resistance indicators in an older male cohort. Similarly, our findings revealed no association between changes in MFF and changes in blood glucose or insulin levels.

Our research, alongside animal model studies (26), indicates a correlation between T1D and an uncoupled bone turnover. The changes in MFF ((ΔMFF_3m–0m_, ΔMFF_1.5m–0m_) in the T1D group positively correlated with serum ΔTRACP5b levels (ΔTRACP5b_3m–0m_, ΔTRACP5b_1.5m–0m_) and osteoclast marker mRNA levels. These findings align with the growing evidence that BMAT acts as a paracrine organ, releasing the pro-osteoclastogenic factor RANKL to drive osteoclastogenesis through direct cell–cell interactions in humans and rodents (35, 36). Supporting this, studies on early-stage diabetic mice demonstrated that elevated bone resorption is primarily mediated by BMAT-derived RANKL rather than bone tissue itself (23). Additionally, co-culture experiments revealed that preadipocytes and osteoclast precursor cells under adipogenic conditions can promote osteoclast differentiation even without exogenous RANKL (37). Together, these findings support the hypothesis that increased BMAT in T1D contributes to heightened osteoclast activity and bone loss.

Contrastingly, earlier studies reported no correlation between MFF and bone turnover biomarkers—including the N-terminal propeptide of type 1 procollagen, parathyroid hormone, β-type I collagen telopeptides, and osteocalcin—in populations such as postmenopausal healthy women, individuals with chronic kidney disease, and those with type 2 diabetes (38–41). This discrepancy may stem from sample heterogeneity, such as prior treatment in chronic kidney disease or type 2 diabetes patients, or methodological differences across studies. The exploration of BMAT as a potential driver of bone loss in T1D has gained momentum, as the decrease in osteoblastogenesis shifts focus toward the role of bone marrow fat. Various murine T1D models have shown increased PPARγ2 expression and higher adipocyte counts in the bone marrow, suggesting a link between T1D and altered BMAT dynamics (23, 42, 43). However, definitive evidence connecting heightened bone marrow fat content to bone loss in T1D remains limited. As such, whether BMAT accumulation directly contributes to bone disorders in T1D remains an open question, warranting further investigation.

A notable strength of our study is its longitudinal design, which allowed for a detailed investigation of the progressive changes in diabetes and their impact on marrow fat content using MR spectroscopy. This approach provided valuable insights into the dynamic relationship between diabetic conditions and marrow fat accumulation. Nevertheless, the study also has limitations. While marrow adipocytes were obtained for molecular analysis, key evaluations—such as the expression of proteins mediating interactions between adipocytes, osteoclasts, and osteoblasts—were not conducted. This limitation underscores the need for further research to unravel the mechanisms by which BMAT influences osteoclast activity, particularly in the later stages of T1D in rabbits. Addressing these gaps will be crucial for advancing our understanding of BMAT’s role in diabetic bone remodeling and its potential implications for therapeutic interventions.

In conclusion, our findings highlight an increased accumulation of BMAT, which is a distinctive feature of diabetic bone, closely associated with elevated bone resorption. This phenomenon is driven by the regulation of mRNA expression of key factors, involving PPARγ2, C/EBPα, FABP4, and osteoclast markers. This regulation could potentially serve as a promising therapeutic target for addressing diabetic osteoporosis.

## Data Availability

The raw data supporting the conclusions of this article will be made available by the authors, without undue reservation.
